# A Breath of Fresh Air in the Fog of Antimicrobial Resistance: Inhaled Polymyxins for Gram-Negative Pneumonia

**DOI:** 10.3390/antibiotics8010027

**Published:** 2019-03-16

**Authors:** Mark Biagi, David Butler, Xing Tan, Samah Qasmieh, Eric Wenzler

**Affiliations:** Department of Pharmacy Practice, College of Pharmacy, University of Illinois at Chicago, 833 South Wood Street, Room 164 (M/C 886), Chicago, IL 60612, USA; mbiagi2@uic.edu (M.B.); dbutler1@uic.edu (D.B.); xt9@uic.edu (X.T.); sqasmieh@uic.edu (S.Q.)

**Keywords:** colistin, polymyxin, HAP, VAP, pneumonia, inhaled antibiotics

## Abstract

Despite advancements in therapy, pneumonia remains the leading cause of death due to infectious diseases. Novel treatment strategies are desperately needed to optimize the antimicrobial therapy of patients suffering from this disease. One such strategy that has recently garnered significant attention is the use of inhaled antibiotics to rapidly achieve therapeutic concentrations directly at the site of infection. In particular, there is significant interest in the role of inhaled polymyxins for the treatment of nosocomial pneumonia, including ventilator-associated pneumonia, due to their retained activity against multi-drug resistant Gram-negative pathogens, including *Acinetobacter baumannii* and *Pseudomonas aeruginosa*. This review will provide a comprehensive overview of the pharmacokinetic/pharmacodynamic profile, clinical outcomes, safety, and potential role of inhaled polymyxins in clinical practice.

## 1. Introduction

Lower respiratory tract infections (LRTIs) are the leading cause of death from infectious diseases and are associated with a substantial economic burden [1,2]. In particular, hospital-acquired pneumonia (HAP) and ventilator-associated pneumonia (VAP) continue to have mortality rates as high as 30% despite advances in medical care and antimicrobial treatment [3,4,5,6,7,8].

Pneumonia is a deep-seated infection of the lung parenchyma that is challenging to treat due to anatomical and physiological barriers that limit the ability of most antimicrobials to adequately reach the site of infection [9]. Pneumonia manifests as a result of microbial pathogens proliferating in the alveolar epithelial lining fluid (ELF) with a subsequent host inflammatory response [10]. Systemically administered antimicrobials must cross several layers of tissue in order to reach the ELF, including the alveolar epithelium, which are connected by tight junctions and fortified with efflux pumps [11,12,13,14]. The amount of drug that reaches the site of infection is further hampered by hypoxia-induced pulmonary vasoconstriction and a chemokine-induced inflammatory response [15]. As such, most systemically administered antimicrobials have limited intrapulmonary penetrance, including commonly used agents like β-lactams and aminoglycosides [9]. Therefore, alternative therapeutic modalities have been increasingly utilized to circumvent these barriers, such as the direct delivery of drug to the lungs via inhalation [16].

The challenge of treating pneumonia is compounded by the increasing rate of resistance among respiratory pathogens without a proportional increase in antimicrobial development [17,18,19]. Increasing resistance is particularly problematic in pneumonia as Gram-negative bacilli represent 8 of the 10 most common organisms isolated from patients hospitalized with pneumonia and inappropriate empiric or definitive antimicrobial therapy are independent predictors of 30-day mortality [20,21]. Gram-negative resistance has become so severe that it has led to the revitalization of previously abandoned agents, such as the polymyxins (polymyxin B and colistin [polymyxin E]). Both agents exhibit similar potent in vitro activity against a wide-spectrum of Gram-negative bacilli, but their utility for the treatment of pneumonia are limited by their low concentrations in ELF after systemic administration, their lack of robust efficacy data, and high toxicity [21,22,23,24]. Chief among these pathogens leading to the revival of the polymyxins are carbapenem-resistant *Pseudomonas aeruginosa*, *Acinetobacter baumannii* (CRAB), and Enterobacteriaceae (CRE) [2,24,25]. As polymyxins remain the most active agents in vitro against these pathogens, they are prime candidates for delivery via alternative routes of administration that may optimize efficacy and minimize toxicity.

Inhalation of antimicrobials capitalizes on the ability to achieve high sustained concentrations of drug in the lungs noninvasively and with minimal systemic exposure, in contrast to parenteral and oral delivery routes. Inhaled therapy is even of greater importance with agents that exhibit dose-limiting toxicities and can allow for administration of therapeutic or even supra-therapeutic doses while minimizing systemic adverse effects [16,26,27]. Moreover, the elevated concentrations achieved in the ELF after inhalation compared to systemic administration alters the application of clinical minimum inhibitory concentration (MIC) breakpoints in the treatment of pneumonia as these breakpoints do not account for the significantly higher concentrations attained at the site of infection through direct delivery to the lungs. With limited penetrance into ELF by most systemic antimicrobials, inhalation is an advantageous route of administration to maximize our current antimicrobial armamentarium [9,16].

The polymyxins remain clinically essential agents due to the limited number of novel antimicrobials in development, the increasing incidence of multi-drug resistant (MDR) Gram-negative pathogens, and the potential for emergence of resistance to new drugs [21,28,29]. This review provides an examination of the pre-clinical and clinical data supporting the use of inhaled polymyxins. It should be noted that the terms “inhaled”, “nebulized”, and “aerosolized” are frequently used interchangeably in the literature. For simplicity, only the term “inhaled” will be used throughout this review. Drug characteristics, available pharmacokinetic (PK) data, pharmacokinetic/pharmacodynamic (PK/PD) parameters, current clinical experience and outcomes, safety and adverse event profiles, and the role of inhaled polymyxins in clinical practice are summarized.

## 2. Pharmacokinetics and Pharmacodynamics of Inhaled Polymyxins

Polymyxin B and colistin methanesulfonate (CMS) exert bactericidal activity against Gram-negative organisms by binding to phospholipids within the cell membrane, leading to disruption in the cell membrane’s permeability, leakage of intracellular contents, and ultimately, cell death [30]. Colistin is commercially available as CMS, an inactive prodrug that is hydrolyzed to colistin sulfate via plasma esterases [31]. This hydrolyzation is often slow and incomplete after intravenous (IV) administration, and similar issues have been demonstrated after inhalation in animals. In two studies conducted in rats to evaluate the conversion kinetics of CMS-to-colistin in ELF versus plasma, a slower and sustained conversion rate in the ELF was observed, leading to higher (but variable) concentrations of colistin in the ELF [32,33]. In the study by Yapa et al., rats were administered CMS (14 mg/kg) either IV or via intratracheal administration. Serial pre- and post-dose blood sampling was performed from 0–4 h in rats receiving IV CMS and from 0–8 h in rats receiving intratracheal CMS [32]. In a second cohort receiving intratracheal CMS, terminal bronchoalveolar lavage (BAL) fluid and a corresponding blood sample was performed at 0.08, 0.5, 2, 4, 6, 8, or 12 h post-dose. Based on population PK modeling the estimated percent of CMS converted to colistin in plasma following IV administration and in ELF following intratracheal administration were 2.6% and 22.6%, respectively. These results were consistent with a previous study also conducted in rats receiving IV or intratracheal CMS (15 mg/kg), in which the estimated percent of CMS converted to colistin in plasma following IV administration and in ELF following intratracheal administration were 12.5% and 39%, respectively [33]. Unfortunately, there are currently no published data evaluating CMS-to-colistin conversion in human ELF. Polymyxin B does not require conversion to an active metabolite and has demonstrated more reliable systemic PK than colistin, which may also translate to inhaled use.

Inhaled polymyxin administration is advantageous compared to parenteral administration for intrapulmonary infections as aerosolized administration achieves higher ELF concentrations helping to achieve bactericidality, prevent resistance, and reduce the likelihood of systemic toxicities [16]. The advantages of the inhaled route are maximized when dosing formulations specifically designed for inhalation are used. For example, optimizing inhaled formulations through advanced particle engineering via jet milling or spray-drying allows for higher delivered doses per inhalation with improved deposition into the lung parenchyma [16]. Conversely, use of parenteral formulations for inhalation has been shown to lead to toxicities related to excipients. Unfortunately, neither polymyxin is available as an inhaled formulation in the U.S. while inhaled colistin (Coly Mycin M^®^) is commercially available in Europe. Furthermore, optimization of inhaled antimicrobial PK requires manipulation of ventilation techniques for patients undergoing mechanical ventilation and being treated for VAP.

Briefly, the ventilator settings should be adjusted to volume control (rather than pressure control) to increase the nebulization efficiency, tidal volumes set to ≥500 mL to increase lung deposition, inspiration-to-expiration ratios increased to prolong inspiratory duration, inspiratory durations set at under 80 L/min (and ideally set at 40 L/min), and nebulization should be synchronized with the flow rate. Since most commercially available nebulizers are not breath synchronized, a ventilator-integrated aerosolization system should be used for mechanically ventilated patients. Finally, although the optimal position of the nebulizer remains unknown, it is often placed 10 to 15 cm from the Y piece of the inspiratory limb and as close as possible to the patient.

To date, the limited data available on the PK of inhaled polymyxins in humans is solely limited to inhaled colistin. Imberti et al. assessed the intrapulmonary concentrations of colistin during BAL in 13 critically ill patients receiving CMS 2 million units (MU) IV every eight hours for at least two days [34]. BAL samples were obtained two hours after the start of CMS infusion. The lower limit of detection was 0.05 mg/L. One patient who received aerosolized CMS (dose not reported) was included as an internal control. Interestingly, this patient was the only one found to have a “relevant” BAL sample concentration (0.48 mg/L), while the patients who received IV CMS were below the lower limit of detection.

**Figure 1:**
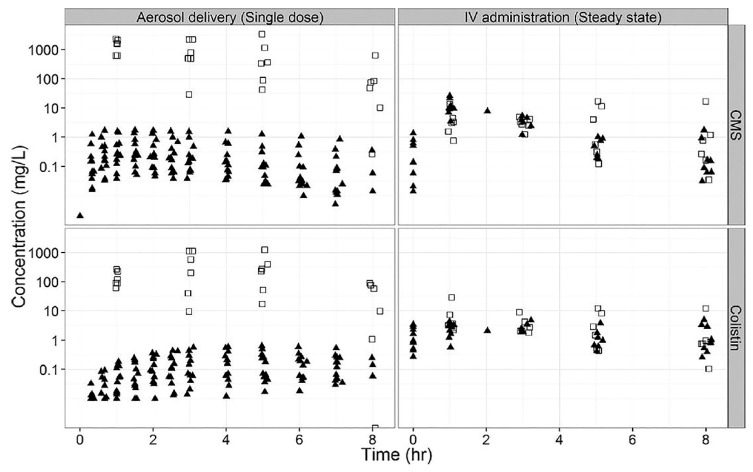
CMS (top panels) and colistin (bottom panels) concentrations in ELF (open squares) and plasma (filled triangles) following a single dose via aerosol or IV administration at steady state. Reprinted with permission from Boisson et al [35].

Boisson et al. evaluated the concentrations of colistin in ELF of 12 critically ill patients who initially received a single dose of inhaled CMS (160 mg dissolved in 10 mL of saline and nebulized over 30 min via vibrating mesh nebulizer [Aeroneb Pro^®^]) followed eight hours later by CMS 160 mg IV every eight hours over 60 min until the end of treatment or therapeutic de-escalation [35]. Following administration of a single nebulized CMS dose, colistin concentrations in ELF ranged from 9.53–1137 mg/L, compared to 1.48–28.9 mg/L following IV administration (Figure 1). Bacterial count predictions over a 24-h time period following simulated administration of either inhaled CMS (2 MU) followed by IV CMS at hours 8 and 16 or IV CMS (2 MU) at 0, 8, and 16 h, were also compared using a wild-type *P. aeruginosa* strain. Assuming a starting inoculum of 10^6^ CFU/mL, the time to “total-kill” was approximately 12 h when the first CMS dose was inhaled, whereas simulated IV CMS only was unable to achieve this target. Additionally, the percentage of bacterial counts below the lower level of quantification (100 CFU/mL) at 24 h following either nebulized or IV administration were 98.5% and 11%, respectively.

Athanassa et al. described the PK of inhaled CMS in 20 mechanically ventilated critically ill patients with VAP [36]. Patients received CMS 80 mg dissolved in 3 mL of half-normal saline every eight hours over 30 min via a vibrating-mesh nebulizer (Aeroneb Pro^®^), which was placed at the inspiratory limb of a non-humidified ventilator circuit, 15 cm away from the Y-piece with fixed ventilator settings. Of note, most isolates were either *A. baumannii* (52.4%) or *P. aeruginosa* (38.1%) with MICs ranging from 0.5–2 mg/L. The median AUC_0-8hELF_ following a single dose was 29.8 mg·h/L (25–75% interquartile range [IQR]: 21.9–54.5 mg·h/L) and the percent of patients with colistin ELF concentrations <2 mg/L (which corresponds to the Clinical & Laboratory Standards Institute [CLSI] susceptibility breakpoint for polymyxins against *A. baumannii* and *P. aeruginosa*) at four and eight hours after administration were 10% and 40%, respectively. For context, recently published polymyxin guidelines recommend targeting a steady-state plasma colistin AUC_24_ of 50 mg·h/L, equating to an average steady-state plasma concentration of ~2 mg/L [37]. However, these same guidelines acknowledge that achieving this plasma AUC_24_ target with IV administration is still highly unlikely to be optimal for the treatment of lung infections. Based on the study by Athanassa et al., it could reasonably be expected that with repeated inhaled dosing, the AUC_0-24hELF_ would exceed 50 mg·h/L [36]. This is important given that the PK/PD index best associated with the efficacy of polymyxins is the *f*AUC/MIC ratio. Theoretically, a higher initial dose with optimized particle engineering, one that may help maintain higher drug concentrations in the ELF over a longer duration, may be necessary for isolates with decreased susceptibility [16,38].

## 3. Clinical Outcomes

The use of inhaled polymyxins for the treatment or prevention of LRTIs has been previously reported with most studies focusing on nosocomial pneumonia (NP), including VAP. Inhaled polymyxins have been evaluated both alone and in combination with IV colistin and/or additional IV agents for the treatment or prevention of NP/VAP.

### 3.1. Colistin Monotherapy

The use of inhaled colistin monotherapy for NP and/or VAP consists primarily of small case series and are summarized in Table 1. Inhaled monotherapy has previously been used to refer to both inhaled colistin with or without additional antibiotics except for IV colistin. For the purposes of this review, inhaled monotherapy will only be used in reference to inhaled colistin without any systemic antibiotics.

In the largest study to date, inhaled colistin monotherapy was prospectively evaluated in patients with VAP due to *A*. *baumannii* or *P. aeruginosa* [39]. Patients infected by β-lactam resistant isolates received inhaled colistin with or without an IV aminoglycoside and patients infected by β-lactam susceptible isolates received an IV β-lactam with or without an IV aminoglycoside. Inhaled colistin was dosed as 5 MU every 8 h and administered over 60 min using a vibrating-plate nebulizer positioned 10 cm proximal to the Y piece, with removal of the heat-moisture exchanger. Clinical outcomes did not significantly differ between treatment groups despite patients in the β-lactam susceptible VAP group receiving appropriate initial therapy at a significantly higher rate (84% vs. 0%, respectively). It should be noted that the dose of inhaled colistin used in this study was significantly higher than previous reports and the nebulization administration technique was optimized. Results of this study provide support for the use of high-dose inhaled colistin for the treatment of MDR *A. baumannii* or *P. aeruginosa* VAP.

### 3.2. Adjunctive Colistin

Inhaled colistin has been extensively studied in combination with IV colistin and/or additional systemic antibiotics with broad-spectrum Gram-negative activity for NP/VAP. Unfortunately, most of this data is drawn from observational and retrospective studies, using various dosing strategies and administration techniques, and there are exceedingly few prospective, randomized, placebo-controlled trials. Studies evaluating the adjunctive use of inhaled colistin for Gram-negative NP/VAP are summarized in Table 2. 

In one of the largest studies to date, 208 patients treated with either IV colistin or inhaled plus IV colistin for Gram-negative VAP due to colistin-only susceptible (COS) isolates were retrospectively matched in a 1:1 ratio [40]. Intravenous colistin was administered every 8–12 h at daily per-kilogram doses of ~100,000 IU, and inhaled colistin was administered as CMS 1 MU three times daily via jet or ultrasonic nebulizer. The primary outcome was clinical cure, defined as resolution of all signs and symptoms of pneumonia and improvement (or lack of progression) of all chest radiograph abnormalities at end of colistin therapy. Patients receiving inhaled plus IV colistin were significantly more likely to have VAP caused by *A. baumannii* (69.2% vs. 53.8%; *p* = 0.02) but significantly less likely to have VAP caused by *Klebsiella pneumoniae* (7.7% vs. 19.2%; *p* = 0.01). The primary endpoint was achieved at a significantly higher rate in the inhaled plus IV colistin group (69.2% vs. 54.8%; *p* = 0.03). Through logistic regression analysis, inhaled plus IV colistin therapy (*p* = 0.009) and trauma-related admissions (*p* = 0.01) were identified as independent predictors of clinical cure.

In another retrospective analysis, 86 patients with monomicrobial COS Gram-negative VAP receiving either IV colistin or inhaled plus IV colistin were matched 1:1 based on age and APACHE II score [41]. Patients with normal renal function received IV colistin 3 MU three times daily with or without inhaled colistin 1 MU twice daily; the nebulizer type was not included. The primary objective was the clinical outcome of VAP, classified as either clinical cure, clinical improvement, clinical failure, or recurrence. Clinical success was classified as either clinical cure or improvement. While no statistically significant differences in outcomes were observed, patients in the inhaled plus IV colistin group had higher rates of clinical cure (54% vs. 32.5%, *p* = 0.05) and clinical success (74% vs. 60%; *p* = 0.10) and a lower all-cause mortality rate (23% vs. 42%; *p* = 0.66). While none of the results reached statistical significance, a trend towards improved clinical outcomes (without an increased risk of toxicity) support the use of adjunctive inhaled colistin for monomicrobial COS Gram-negative VAP. However, key limitations of this study including omission of details regarding the timing of colistin therapy initiation, receipt of concomitant antibiotics, and cumulative colistin dose between treatment groups should be noted and have been previously reviewed in detail elsewhere [42,43].

In the largest study to date, Korkmaz-Ekrin et al. conducted a multicenter, retrospective study evaluating patients with MDR *A. baumannii* or *P. aeruginosa* HAP treated with either IV colistin (*n* = 210) or inhaled plus IV colistin (*n* = 69) [51]. The colistin dosing regimens and administration technique of inhaled colistin were not described. Additionally, >80% of patients received additional IV antibiotics, but no further details were provided. Rates of clinical response and microbiological eradication (among patients with follow-up microbiologic data) were significantly higher among those treated with inhaled plus IV colistin (66.7% vs. 47.6%; *p* = 0.008 and 59.4% vs. 41%; *p* < 0.001, respectively). Many limitations were present in this brief letter to the editor as several key pieces of information were not reported, but results of this study were consistent with those found in a previous meta-analysis [77]. 

In a retrospective study of 123 patients diagnosed with *A. baumannii* NP (*n* = 40) or VAP (*n* = 83), patients were divided based on whether they received IV colistin (*n* = 80) or inhaled plus IV colistin (*n* = 43) [52]. Additional systemic therapy was co-administered in 97.6% of patients. Intravenous colistin was administered as 150 mg colistin base activity (CBA) every 12 h and inhaled colistin was administered as 75 mg of CBA via nebulizer every 12 h; the nebulizer type was not described. Patients receiving inhaled colistin were significantly less likely to have respiratory failure at baseline (14% vs. 32.5%; *p* = 0.025). There were no significant differences noted in the rates of clinical success (37.2% vs. 37.5%; *p* = 0.974), eradication (46.5% vs. 50%; *p* = 0.712), persistence (44.2% vs. 37.5%; *p* = 0.470), 30-day mortality (53.5% vs. 47.5%; *p* = 0.526), or nephrotoxicity (48.8% vs. 53.8%; *p* = 0.603) among patients receiving IV colistin with or without inhaled colistin, respectively. Select limitations include the lack of an assessment of disease severity, an inhaled colistin dose lower than used in some other studies, and no description regarding the rates of appropriate empiric therapy or time from positive culture to colistin therapy initiation.

While many studies of adjunctive therapy have evaluated inhaled colistin in the setting of co-administration with IV colistin, few studies have evaluated adjunctive inhaled colistin with other systemic antibiotics. In one of the few prospective, randomized trials evaluating the safety and efficacy of inhaled colistin, 149 critically ill patients with Gram-negative VAP were randomly assigned to empirically receive either IV colistin plus imipenem (*n* = 76) or inhaled colistin plus imipenem (*n* = 73) [53]. Colistin was either administered via nebulization as CMS 4 MU three times daily over 30 min via an ultrasonic vibrating plate nebulizer or as a single IV CMS 9 MU loading dose followed by 4.5 MU twice daily (or renally dose-adjusted equivalent). Following finalization of the susceptibility report, COS isolates were treated with targeted colistin monotherapy while isolates susceptible to additional agents were treated with targeted therapy consisting of inhaled or IV colistin (depending randomization) plus a β-lactam, aminoglycoside, or tigecycline. The primary outcome was cure of VAP at day 14, defined as clinical pulmonary infection score <6 and bacteriological eradication. The most common causative pathogens isolated were *A. baumannii* (*n* = 68) and *P. aeruginosa* (*n* = 26). There were no significant differences observed in the rates of clinical cure, length of mechanical ventilation, ICU length of stay, or 28-day all-cause mortality between treatment groups. However, patients receiving inhaled colistin had a significantly decreased time to bacteriological eradication (9.89 vs. 11.26 days; *p* = 0.023), weaning from mechanical ventilation among ICU survivors (13 vs. 18 days; *p* = 0.012), incidence of acute renal failure (17.8% vs. 39.4%; *p* = 0.004), and a significantly improved P/F ratio at day 14. The authors concluded that inhaled colistin was as effective as IV colistin and suggested that inhaled colistin be considered a first-line therapy for VAP due to MDR Gram-negative bacilli in patients without septic shock and/or bacteremia.

In a separate study, the outcomes of 219 patients treated with systemic antibiotics in addition to either IV colistin (*n* = 93) or inhaled colistin (*n* = 126) for carbapenem-resistant *A. baumannii* (CRAB) VAP were retrospectively evaluated [54]. All patients received IV or inhaled colistin for ≥3 days and colistin therapy was initiated within five days before or after the date of index culture. Inhaled colistin was administered over 30 min via a conventional jet nebulizer and doses ranged from 75 mg CBA every 12 h to 150 mg CBA every 8 h. The IV colistin dose was 2.5 mg/kg CBA twice daily in patients with creatinine clearance >80 mL/min and was adjusted for patients with renal impairment as described. Patients receiving inhaled colistin were significantly older (70 vs. 65 years; *p* = 0.010) and more likely to receive concomitant susceptible systemic antibiotics (55% vs. 17%; *p* < 0.001) but significantly less likely to have chronic kidney disease (6% vs. 14%; *p* = 0.032) or septic shock (45% vs. 62%; *p* = 0.012). No difference in microbiologic failure was observed between treatment groups but patients receiving inhaled colistin had a significantly lower rate of clinical failure (39% vs. 57%; *p* = 0.008), ICU mortality (40% vs. 59%; *p* = 0.006), and acute kidney injury (AKI) during colistin therapy (16% vs. 38%; *p* < 0.001). In univariate analysis, patients who received inhaled colistin doses of <300 mg CBA/day (*n* = 49) had a trend towards an increased clinical failure rate (OR 1.74; 95% CI, 0.83–3.61; *p* = 0.14).

Thirty-nine patients from each group of the previous study were subsequently matched by propensity score [54]. Likewise, no significant differences for patients receiving either inhaled or IV colistin were noted regarding the rates of clinical failure (36% vs. 54%; *p* = 0.11), microbiologic failure (51% vs. 49%; *p* = 0.82), or ICU mortality (36% vs. 56%; *p* = 0.07). In multivariate analysis, risk factors independently associated with clinical failure were medical ICU admission (aOR, 7.14; 95% CI, 1.60–32.00, *p* = 0.010) and septic shock (aOR, 3.93; 95% CI, 1.27–12.17; *p* = 0.018). Altogether, results of this study suggest that inhaled colistin in combination with active systemic antibiotics other than IV colistin may be a clinically useful treatment for CRAB VAP and support the notion that inhaled colistin doses may need to be intensified during therapy to improve clinical outcomes.

## 4. Safety

### 4.1. Nephrotoxicity

The potential for nephrotoxicity is one of the most common and clinically important concerns of polymyxin therapy. Following IV administration, the reported incidence of nephrotoxicity has varied widely (0–70%) owing in part to study design, heterogeneous definitions of nephrotoxicity, and patient-specific factors [78,79,80,81,82,83]. In studies using standardized definitions for nephrotoxicity (i.e., RIFLE or Acute Kidney Injury Network [AKIN]) incidence of any AKI ranged from 20–60% [73,82,83,84]. Current evidence suggests that the rate of nephrotoxicity is lower with polymyxin B than colistin, with colistin identified as an independent risk factor for renal failure [84,85]. Although practice varies substantially, clinician perceptions mirror this disparity in nephrotoxicity [86], which may be driven by more severe nephrotoxicity with colistin (“F” in RIFLE) [84], earlier onset to nephrotoxicity with colistin compared to polymyxin B [85], or due to the extensive renal clearance of CMS but not colistin or polymyxin B [35,87,88,89,90].

Like studies of IV polymyxins, trials of inhaled polymyxin therapy are often plagued with heterogeneity of patients, doses, and definitions, which creates a challenge in differentiating the true incidence and cause of nephrotoxicity. As previously discussed, inhaled therapy minimizes systemic exposure and therefore subsequent renal toxicities. In practice, the incidence of AKI in patients treated with inhaled polymyxin therapy usually reflects the population studied, and has ranged from 0% to 30%, with the highest rates occurring in the most critically ill patients [77,91,92,93,94]. In a randomized controlled trial of inhaled versus IV CMS for Gram-negative VAP, inhaled CMS had a significantly lower incidence of nephrotoxicity compared to IV administration (17.8% vs. 39.4%, *p* = 0.004). Although not administered as monotherapy, stratified analyses of these well-matched groups revealed that concomitant nephrotoxic agents were associated with increased renal dysfunction with IV, but not inhaled, CMS [53]. A separate randomized placebo-controlled trial compared adjunctive inhaled CMS versus inhaled saline in patients with Gram-negative VAP, administering 2.25 MU of CMS or saline placebo twice daily via jet or ultrasonic nebulizer for the duration of IV antibiotic treatment. This study is the most rigorous placebo-controlled trial to date with inhaled polymyxins, including 100 critically ill patients, and in comparison to inhaled normal saline, inhaled CMS did not increase the rates of AKI (22.4% vs. 25.5%; *p* = 0.82) [56].

Studies with IV polymyxins report that overall exposure (e.g., duration of therapy, daily dose by ideal body weight, minimum plasma concentrations) are independently associated with nephrotoxicity [79,81,95,96,97]; however, nephrotoxicity from inhaled therapy does not appear to be dose-related [39,53,93]. Studies evaluating inhaled colistin at daily doses of 4 MU showed similar nephrotoxicity (0–38%) [93] as studies with substantially higher daily doses of 12 MU (17.8%) [53] and 15 MU (12%) [39]. Indeed, as plasma concentrations following inhaled polymyxin therapy rarely peak above 1 mg/L [35,98,99,100], and IV dosing strategies often target an AUC_24_ of 60 mg·h/L (C_ss,avg_ 2.5 mg/L) [101], systemic exposure due to inhaled therapy is likely to be to less than 1% that of equivalent IV therapy. Taken together, inhaled polymyxins are expected to have increased safety and efficacy compared to IV therapy through increased drug exposure in the ELF and decreased systemic exposure, thus shifting the benefit-risk ratio in favor of inhaled therapy.

### 4.2. Bronchoconstriction

Another concern of inhaled polymyxins is the reported risk of bronchospasm and bronchoconstriction, particularly in patients with cystic fibrosis (CF) or other chronic airway disease [82,102,103,104,105,106]. The mechanism behind bronchoconstriction is unknown, but is thought to be caused by chemical irritation of the airways and histamine release [107]. Indeed, the polypeptide structure of polymyxins seems to induce mast cell degranulation in vitro, with polymyxin B having more potency than colistin [108]. Corollary to this is the potential for direct toxicity to the pulmonary epithelium. One study examined the mechanism and direct toxicity of polymyxins on human lung epithelial cells in vitro and found that active polymyxins (i.e., polymyxin B and colistin, but not CMS) induce mitochondrial oxidative stress that lead to concentration- and time-dependent inflammation and apoptosis at concentrations >2000 mg/L [109]. This in vitro data suggests an upper threshold for colistin and polymyxin B concentrations in the ELF, above which the polymyxins may be locally toxic. A recent murine study of colistin dry powder aerosols in healthy rats showed ELF colistin concentrations reaching 607 mg/L induced minor lung inflammation at 24 h post dose [110]. A similar murine lung infection model demonstrated that while aerosolized polymyxin B at ELF concentrations reaching 184 mg/L induced minor inflammation in the lungs of healthy mice, it significantly reduced the pulmonary inflammation sustained by *P*. *aeruginosa* pneumonia in neutropenic mice [111]. As maximal ELF concentrations from either human or animal studies rarely exceed 1000 mg/L [35,100,110,111,112], and total daily doses as high as 15 MU of CMS have been well tolerated [93], therapeutic doses are unlikely to be directly toxic to the pulmonary epithelium.

Conversely, in children and adults with CF, inhaled CMS may result in a transient decrease in respiratory function and increase in perceived chest tightness [104,113,114]. Bronchoconstriction is more likely with inhaled colistin sulfate than CMS, as was shown in a randomized double-blind study in CF patients where treatment with inhaled colistin sulfate lead to significantly decreased measurements of respiratory function compared to CMS [115]. In patients without CF, recent estimates from clinical trials show the incidence of bronchospasm to be less than 10% following inhaled CMS [56,116], and possibly as high as 15–20% following inhaled polymyxin B [117,118]. In patients with underlying pulmonary disease, clinicians should consider administering bronchodilators immediately prior to inhaled polymyxins, both to increase the drug deposition in the terminal alveoli and decrease the risk of bronchospasm [16,104].

While rare, more severe toxicities have also occurred with inhaled polymyxin therapy including acute respiratory distress syndrome (ARDS) and eosinophilia [105,106,119]. This serves as a reminder that clinicians should consider a monitored trial dose, especially in those with severe lung disease. Additionally, extended delays between reconstitution and administration has been implicated in ARDS. Stability data shows that more than 60% of the prodrug can convert to its active form and other degradation products within 48 h following reconstitution [120], and the US Food and Drug Administration has advised that storing for longer than 24 h increases the potential for lung toxicity [121].

### 4.3. Resistance

In addition to toxicities, a historical concern with the use of inhaled polymyxins is the development of bacterial resistance. The first argument concerns the development of an antibiotic gradient from the proximal to distal airways due to the deposition patterns of inhaled antibiotics; however, this notion should no longer be a concern for several reasons. First, the concern is not that a drug concentration gradient in the lungs promotes resistance development, but the theoretical concern of subinhibitory concentrations in the deep airways. Through a better understanding of the particle characteristics and inhalation techniques, in conjunction with more efficient delivery devices, drugs can be reliably delivered in effective concentrations to the distal airways therefore minimizing or eliminating this purported gradient. Additionally, through mathematical modeling and a more complete understanding of PK/PD parameters, the concept of the mutant prevention concentration (MPC) was introduced which represents the concentration at which a drug severely restricts the selection of resistant mutants. Usually several-fold higher than the MIC, the MPC requires substantially higher drug exposures to achieve. As elucidated in the previous sections, administering polymyxins via inhalation achieves concentrations in the lungs exponentially higher than after IV administration. Therefore, treatment with inhaled polymyxins will more reliably achieve concentrations exceeding the MIC and MPC at the site of infection compared to after IV administration. Recent in vitro analyses have shown that the MPC_90_ of polymyxins for *A. baumannii, K. pneumoniae*, and *P*. *aeruginosa* ranged from 64 mg/L to >128 mg/L [122,123,124,125]. These concentrations far exceed those found in the ELF following IV administration, which are often below the limits of quantitation [98,100,110,111], but can reliably be achieved following properly aerosolized therapy which is expected to attain concentrations exceeding 100 mg/L [35,98,99,100,110,111,112].

Sporadic cases of acquired colistin resistance or decreased susceptibility associated with inhaled therapy have been previously reported [39,46,47,119]. In the study by Hsieh et al., a two-fold MIC increase was observed in 5/28 (17.8%) patients, including one patient who developed colistin resistance [47]. Choi et al. reported the development of colistin resistance in 3/12 (25%) of patients with a median interval to resistance development of 7 days (range 5–19); however, despite the development of resistance, all three patients had favorable clinical outcomes with continued inhaled colistin therapy [46].

Similarly, the emergence of opportunistic organisms with intrinsic resistance to polymyxins has been noted, such as *Proteus* spp. or *Elizabethakingae* spp. [126,127,128]; however, these are of questionable clinical relevance to pneumonia. Admission to an acute care hospital was unsurprisingly found to be a major risk factor for emergence of these intrinsically resistant microorganisms [119], which stresses the importance of appropriate infection control and minimizing prolonged courses [129,130,131,132]. Additional care should be taken to properly clean and dry nebulizers, as nebulizer reservoirs may serve as cesspools for growth and direct aerosolization of hydrophilic Gram-negative organisms [133]. The use of closed-loop or filtered-exhaust nebulizer systems are also highly recommended as unfiltered open-exhaust nebulizers can cause environmental contamination with antibiotics, promoting resistance [134].

## 5. Discussion

Despite advancements in therapy, NP and VAP remain common complications of hospitalization and are associated with significant morbidity and mortality [135,136,137,138,139]. Inhaled polymyxins are important therapeutic options for NP/VAP due to their retained activity against MDR Gram-negative pathogens, including *A. baumannii* and *P. aeruginosa* [140,141]. Despite their availability for decades, polymyxins have been only sparingly used due to concerns of nephrotoxicity and neurotoxicity. Administration through an inhaled route delivers high local concentrations directly to the site of infection, which would otherwise be unobtainable following IV administration, and minimizes the toxicities encountered with IV administration. This has particularly important implications considering that patients with NP/VAP tend to be critically ill and already receiving other nephrotoxic agents.

Although anecdotal data may support the use of inhaled colistin as monotherapy for Gram-negative NP/VAP, further data are needed to support the routine use of inhaled polymyxin monotherapy. Potential candidates for inhaled polymyxin monotherapy are non-critically ill patients with a documented Gram-negative pneumonia who are hemodynamically stable and without extrapulmonary signs or symptoms of infection. Adjunctive therapy with inhaled colistin should be reserved for patients with confirmed or suspected NP/VAP due to MDR Gram-negative pathogens, particularly *A. baumannii* and *P. aeruginosa*. Until further data is available, inhaled polymyxins should be continued for the same duration as systemic antibiotics.

Various doses and frequencies of inhaled colistin have been previously studied (primarily retrospectively), but the optimal dosing regimen remains unknown. Anecdotal data suggests higher inhaled colistin doses are associated with improved efficacy without significantly increasing the risk of adverse events, although the importance of the delivery system and administration technique cannot be overstated. At minimum, we recommend an inhaled CMS doses of 80 mg twice daily with consideration for higher doses in patients not responding to therapy.

## 6. Conclusions

Despite these presumed advantages of inhaled polymyxins, there are several remaining questions and limitations of current literature. Future randomized, prospective trials are needed to determine the optimal formulation, dose, administration technique, frequency, and duration of inhaled polymyxins as current studies are primarily limited to retrospective studies using widely variable dosing regimens that primarily employ the parenteral formulation. Finally, the development of alternative polymyxin-derived agents such as liposomal colistin and polymyxin analogues may help further improve the efficacy and minimize the toxicity of inhaled polymyxins. 

## Figures and Tables

**Table 1:** Summary of studies evaluating inhaled colistin without concomitant IV colistin for Gram-negative nosocomial pneumonia and/or ventilator-associated pneumonia.

Ref	Study Design	Patient Population	Comparator Group(s)	Inhaled Group(s)	Duration (Days) ^A^	Clinical Outcome(s)	Toxicity	Comments
[39]	Prospective, observational	MDR VAP caused by *A. baumannii* or *P. aeruginosa* (*n* = 165)	MDR VAP: Inhaled colistin + IV aminoglycoside (*n* = 15)Susceptible VAP: IV β-lactam + IV aminoglycoside or fluoroquinolone (*n* = 122)	5 MU q8h (*n* = 28)	12 ^B^	No significant differences in clinical outcomes between patients with MDR VAP treated with inhaled colistin with or without IV aminoglycoside and patients with susceptible VAP	Serum creatinine remained stable during treatment in both groups	All patients in MDR VAP group received inappropriate empiric therapy
[44]	Retrospective	MDR NP caused by COS *A. baumannii* or *P. aeruginosa* (*n* = 21)	None	1 MU BID (*n* = 19) 1 MU TID (*n* = 1) 1 MU QID (*n* = 1)	14 (2–36)	Clinical cure or improvement: 57.1% Microbial eradication:Documented: 52.4% Presumed: 33.3% All-cause mortality: 47.6% Attributable mortality: 14.3%	Bronchoconstriction: (*n* = 1; 4.8%)	Seven patients cured of pneumonia subsequently died of underlying and unrelated conditions Most patients received concomitant antibiotics for additional extrapulmonary sites of infection
[45]	Retrospective	NP/VAP (*n* = 5)	None	1 MU q8h (*n* = 4) 500,000 U q6h (*n* = 1)	7.2	Clinical success: 80% Mortality: 20%	NR	All patients received additional systemic antibiotics that were resistant
[46]	Retrospective	COS *A. baumannii* pneumonia (*n* = 12)	None	75 mg BID ± systemic antibiotics	17 (5–31)	Clinical cure or improvement: 83.3% Microbial eradication: 50% All-cause in-hospital mortality: 33.3%	Hypersensitivity: (*n* = 1; 8.3%)	Most patients received concomitant systemic antibiotics and all patients received other broad-spectrum antibiotics prior to initiation of colistin Three isolates developed colistin resistance
[47]	Retrospective	XDR *A. baumannii* pneumonia (*n* = 38)	Inhaled colistin + IV tigecycline (*n* = 29)	2 MU BID (*n* = 9)	13.5 ± 6.5 ^C^	No significant difference in clinical outcomes, microbiological eradication, or 30-day mortality	Bronchospasm: (*n* = 2; 1.7%) ^D^	Isolates in the inhaled colistin only group were significantly more likely to be tigecycline-resistant
[48]	Retrospective	*A. baumannii* VAP (*n* = 31 preterm infants)	Systemic antibiotics (*n* = 23)	80 mg q12h (*n* = 8)	9.1 (4–22)	All patients in both groups were cured and subsequently discharged	NR	Four pre-term infants in the inhaled colistin received active systemic antibiotics for 12–21 days without improvement prior to inhaled colistin monotherapy
[49]	Retrospective	MDR *A. baumannii* pneumonia or colonization (*n* = 135)	Tigecycline (*n* = 40) Ampicillin/sulbactam (*n* = 12) No therapy (*n* = 2)	2 MU BID (*n* = 54) 2 MU BID + systemic antibiotics (*n* = 27)	NR	Significantly higher eradication rate in patients receiving inhaled colistin monotherapy compared to IV therapy only (61.1% vs. 29.6%; *p* = 0.001) No significant difference in 28-day or in-hospital mortality	NR	Patients receiving inhaled colistin were significantly more likely to have colonization 10/37 patients who failed to achieve microbiological eradication at day 14 were eradicated at day 28 with prolonged use of inhaled colistin
[50]	Retrospective, case-controlled	XDR *A. calcoaceticus-A baumannii* complex pneumonia (*n* = 212)	Inhaled colistin + tigecycline (*n* = 106)	2 MU TID (*n* = 106)	12.2 ± 6 ^E^	No difference in 30-day mortality	Bronchospasm: (*n* = 4; 1.9%)	Majority of patients received systemic antibiotics prior to enrollment

**Abbreviations**: MDR: multidrug-resistant; VAP: ventilator-associated pneumonia; q8h: every 8 h; NP: nosocomial pneumonia; COS: colistin-only susceptible; BID: twice daily; TID: three times daily: QID: four times daily; q6h: every 6 h; NR: not reported; XDR: extensively drug-resistant; q12h: every 12 h; ^A^ Presented as means, means ± standard deviations, or median (range); ^B^ Reported for all patients receiving inhaled colistin with or without an aminoglycoside (*n* = 43); ^C^ Reported for all patients with pneumonia who received inhaled colistin with or without systemic antibiotics (*n* = 57); ^D^ Reported for all patients with pneumonia or colonization who received inhaled colistin with or without systemic antibiotics (*n* = 118); both episodes of bronchospasm occurred in patients with underlying chronic obstructive pulmonary diseases; ^E^ Reported for all patients receiving inhaled colistin with or without tigecycline (*n* = 212).

**Table 2:** Summary of studies evaluating inhaled colistin as adjunctive therapy to IV colistin for Gram-negative nosocomial pneumonia and/or ventilator-associated pneumonia.

Ref	Study Design	Patient Population	Comparator Group(s)	Inhaled Group(s)	Duration (days) ^A^	Clinical Outcome(s)	Inhaled Toxicity	Comments
[40]	Retrospective, matched	COS VAP (*n* = 208)	IV colistin (*n* = 104)	1 MU q8h + IV colistin (*n* = 104)	7 (5–14) ^C^	Improved clinical cure in inhaled-antibiotic group; no significant difference in microbiological cure	NR	Concomitant systemic antibiotics not described
[41]	Retrospective, matched	MDR VAP (*n* = 86)	IV colistin (*n* = 43)	1 MU q12h + IV colistin (*n* = 43)	13 (5–56) ^F^	Improved clinical cure in group administered inhaled antibiotic; no difference in microbiological eradication	NR	No detail provided on concomitant systemic antibiotics
[51]	Retrospective	MDR *A. baumannii* or *P. aeruginosa* HAP (*n* = 279)	IV colistin ± systemic antibiotics (*n* = 210)	Inhaled and IV colistin ± systemic antibiotics (*n* = 69) ^E^	NR	Significantly higher clinical response and eradication rates with inhaled therapy No difference in mortality with inhaled therapy	No significant difference in rate of nephrotoxicity	No data on dose or duration of inhaled colistin therapy Most patients (82.8%) received concomitant systemic antibiotics
[52]	Retrospective, case-controlled	*A. baumannii* NP or VAP (*n* = 123)	IV colistin + systemic antibiotics (*n* = 80)	75 mg q12h + IV colistin + systemic antibiotics (*n* = 43)	11.2 ± 6 ^C^	No differences in clinical success, microbiological eradication, or mortality	No significant difference in rate of nephrotoxicity	No assessment of disease severity
[53]	Prospective, randomized	VAP (*n* = 149)	IV colistin + imipenem (*n* = 76)	4 MU TID + imipenem (*n* = 73)	≥14 ^B^	No significant difference in clinical cure; decreased time to microbiological eradication with inhaled therapy	Bronchospasm: (*n* = 2; 2.7%) Neurotoxicity: (*n* = 9; 12.3%) ^I^	Clinical cure rates in a subset of patients receiving colistin monotherapy were 84% and 58% (*p* = 0.20) for inhaled and IV monotherapy, respectively
[54]	Retrospective	CRAB VAP (*n* = 219)	IV colistin + systemic antibiotics (*n* = 57) IV colistin (*n* = 36)	75 mg q12h − 150 mg q8h + systemic antibiotics (*n* = 104) 75 mg q12h to 150 mg q8h (*n* = 22)	17 (10–25)	Significantly lower rates of clinical failure, ICU mortality, and AKI with inhaled colistin	NR	Patients receiving inhaled colistin were significantly more likely to receive active concomitant antibiotics and less likely to have septic shock
[55]	Prospective, observational	MDR VAP (*n* = 60)	None	1 MU TID + systemic antibiotics ^B^	16.4 ± 10.9	Clinical improvement: 83.3% All-cause mortality: 25%	NR	No comparator group; microbiological data not provided
[56]	Randomized, placebo controlled	VAP (*n* = 100)	Placebo inhalation + systemic antibiotics (*n* = 49)	75 mg q12h + systemic antibiotics (*n* = 51)	9.5 ± 4.6	No difference in clinical outcome; improved microbiological outcome with inhaled colistin	Bronchospasm: (*n* = 4; 7.8%)	Clinical and microbiological outcomes evaluated at 28 days
[57]	Retrospective	MDR *A. baumannii* VAP (*n* = 45)	IV colistin (*n* = 15)	75 mg BID + IV colistin (*n* = 29)	14	No difference in clinical or microbiological outcome at day 5 or end of therapy	NR	87% of patients with severe sepsis/septic shock at baseline
[58]	Retrospective	MDR *P. aeruginosa* NP (*n* = 20)	IV colistin + β-lactam (*n* = 5)	2 MU TID + β-lactam (*n* = 6) 2 MU TID + IV colistin + β-lactam (*n* = 9)	IV + inhaled: 19.3 (3–46) Inhaled alone: 27.2 (6–96) IV alone: 21.0 (9–28)	78% favorable response with IV + inhaled vs. 100% for inhaled alone and 40% for IV alone; no patients achieved microbiological eradication	NR	56% of patients on IV and inhaled colistin also had extrapulmonary infection
[59]	Retrospective	MDR *A. baumannii* VAP (*n* = 45)	None	Mean daily dose of 4.29 MU + IV colistin + systemic antibiotics	10.29	Favorable clinical outcome: 57.8% Microbiological eradication: 37.8%	NR	Only 60% had follow-up cultures available
[60]	Retrospective, multicenter	MDR NP (*n* = 95)	IV colistin + systemic antibiotics (*n* = 51)	75 or 150 mg q12h + systemic antibiotics (*n* = 44)	11 (7–16.25)	No significant difference in clinical cure, microbiological eradication, or mortality	NR	Clinical cure rate higher in group administered inhaled antibiotic when only patients with high quality respiratory cultures were evaluated
[61]	Prospective, observational	MDR *P. aeruginosa* VAP (*n* = 8)	None	500,000 IU q8h + IV colistin	15.9 ^D^	Clinical cure: 70% ^E^	NR	Six patients had concomitant co-infections
[62]	Retrospective	Pneumonia (*n* = 49)	None	500,000 IU q6h + systemic antibiotics	12 ± 8	Microbiological eradication: 93%	NR	Parenteral formulation used for inhalation Concomitant antibiotics not described
[63]	Retrospective	VAP (*n* = 121)	IV colistin + systemic antibiotics (*n* = 43)	2.1 MU per day + IV colistin + systemic antibiotics (*n* = 78)	16.9 ± 9.8 ^G^	Significantly improved clinical cure in group administered inhaled antibiotic; no difference in mortality	NR	Significantly more patients in group administered IV antibiotic only with COS organisms; use of inhaled colistin was independent predictor of clinical cure
[64]	Retrospective	MDR NP (*n* = 8)	None	0.5 MU q6h- 2 MU q8h + IV colistin and/or systemicantibiotics	8.9 (3–19)	Clinical improvement or cure: 87.5% Bacterial eradication: 80%	NR	No uniform inhaled dosing strategy or duration
[65]	Retrospective	MDR pneumonia (*n* = 29)	IV colistin ± systemic antibiotics (*n* = 6) Inhaled colistin ± systemic antibiotics (*n* = 6)	Inhaled and IV colistin ± systemic antibiotics (*n* = 17) ^H^	NR	Survival rates were 41.1% in patients receiving both inhaled and IV colistin compared to 100% with inhaled colistin only and 66.7% with IV colistin only	NR	Patients in the IV + inhaled colistin group may have had additional sites of infection Most patients were reported to have received additional systemic antibiotics
[66]	Retrospective	*A. baumannii* NP or tracheobronchitis (*n* = 31)	IV colistin ± systemic antibiotics (*n* = 14) Inhaled colistin ± systemic antibiotics (*n* = 7)	500,000 IU q6h-1 MU q8-12h +IV colistin ± systemic antibiotics (*n* = 10)	NR	Microbiological eradication: Inhaled only: 100% IV only: 42.9% Inhaled + IV: 66.7%	NR	16/31 patients were diagnosed with tracheobronchitis Additional systemic antibiotics not fully described
[67]	Prospective	COS VAP (*n* = 9)	None	1 MU q12h + IV colistin ± systemic antibiotics	13 ± 6.5	Clinical cure or improvement: 77.8% All-cause in-hospital mortality: 22.2%	NR	No specific information regarding additional systemic antibiotics administered to patients with VAP
[68]	Prospective	MDR NP (*n* = 40)	IV colistin (*n* = 12)	2 MU q12h + IV colistin (*n* = 28)	12–15	Clinical failure and mortality significantly higher in the IV colistin group	NR	Concomitant systemic antibiotics not addressed
[69]	Retrospective	MDR VAP (*n* = 31)	IV colistin ± systemic antibiotics (*n* = 23)	2 MU BID + IV colistin (*n* = 8)	10.3 ± 5.72 ^C^	Microbiological eradication significantly higher in the inhaled group No difference in ICU mortality	Bronchoconstriction: (*n* = 1; 12.5%)	Some patients also had extrapulmonary sites of infection Concomitant systemic antibiotics not described
[70]	Retrospective, matched case-control	*A. baumannii* NP or colonization (*n* = 78)	Systemic antibiotics (*n* = 39)	2 MU BID + systemic antibiotics (*n* = 32) 2 MU BID (*n* = 7)	10.9 ± 3.6	Significantly higher 14-day microbiological eradication with inhaled colistin No difference in mortality	No significant differences in incidence of hemodynamic instability, need for intubation, or nephrotoxicity	All isolates were only susceptible to colistin, tigecycline, or sulbactam
[71]	Observational cohort	COS *A. baumannii* VAP (*n* = 16)	None	1 MU TID + IV rifampicin	15	All patients had clinical and microbiological success	NR	Three patients had concomitant bacteremia Therapy was initiated based on culture results and lack of response to empiric regimen
[72]	Prospective, randomized	VAP (*n* = 102)	Systemic antibiotics (*n* = 50)	1 MU q8h + systemic antibiotics (*n* = 52)	5 ^B^	Significantly improved rate of favorable outcomes, 30-day mortality, and clearance of MDR pathogens in inhaled colistin group	NR	Inhaled colistin given only 5 days
[73]	Retrospective	MDR VAP (*n* = 25)	None	75 mg QID − 150 mg BID ± systemic antibiotics ^B^	11.7 ± 7.1	In-hospital mortality: 40% Microbiological eradication: 84.6% (11/13)	Patients receiving >2 concomitant nephrotoxins significantly more likely to develop AKI	Concomitant systemic antibiotics not fully described
[74]	Retrospective	MDR *A. baumannii* VAP (*n* = 95)	IV colistin ± systemic antibiotics (*n* = 44)	4.5 MU q8h ± systemic antibiotics (*n* = 51)	12.6 ± 6.1	No significant differences between in clinical cure, microbiological eradication, or infectious mortality	Nephrotoxicity significantly lower with inhaled colistin: (15.7% vs. 60.5%; *p* < 0.00001)	Patients receiving inhaled colistin were more likely to be older and have higher APACHE II scores
[75]	Prospective, case-controlled	COS VAP (*n* = 40)	Systemic antibiotics (*n* = 20)	1 MU q12h + systemic antibiotics (*n* = 20)	5 ^B^	No significant differences in clinical cure or mortality Microbiological eradication significantly higher in patients receiving inhaled colistin	NR	Inhaled colistin only administered for 5 days
[76]	Prospective, randomized, controlled	VAP (*n* = 50)	Systemic antibiotics (*n* = 25)	2 MU q8h + systemic antibiotics (*n* = 25)	5	Significantly higher microbiological eradication rate at day 5 with inhaled therapy	NR	Systemic antibiotics not described Causative organisms were unevenly distributed among the treatment groups

**Abbreviations**: MDR: multidrug-resistant; VAP: ventilator-associated pneumonia; NR: not reported; q12h: every 12 h; COS: colistin-only susceptible; q8h: every 8 h; BID: twice daily; ICU: intensive care unit; NP: nosocomial pneumonia; TID: three times daily; q6h: every 6 h; HAP: hospital-acquired pneumonia; QID: four times daily; AKI: acute kidney injury; CRAB: carbapenem-resistant *Acinetobacter baumannii*; ^A^ Presented as means, means ± standard deviations, medians (interquartile ranges [underlined]), means (ranges), or (ranges) unless otherwise specified.; ^B^ As stated in methods; ^C^ Reported for IV/inhaled group; ^D^ As reported for IV therapy only. Inhaled therapy was continued until eradication.; ^E^ *n* = 10; ^F^ Presented as median (range). ^G^ As reported for IV colistin therapy only. All patients received inhaled colistin for at least 3 days or more than 50% of the duration of the treatment with IV colistin. ^H^ Inhaled colistin dose note reported. ^I^ The authors report the causality to colistin was uncertain due to co-administration of other neurotoxic agents (narcotics, sedatives, steroids).
